# ADEPT, a dynamic next generation sequencing data error-detection program with trimming

**DOI:** 10.1186/s12859-016-0967-z

**Published:** 2016-02-29

**Authors:** Shihai Feng, Chien-Chi Lo, Po-E Li, Patrick S. G. Chain

**Affiliations:** Genome Science Group, Bioscience Division, Los Alamos National Laboratory, Los Alamos, NM 87545 USA

**Keywords:** Next generation sequencing, Illumina error prediction, Local quality scores, Position-specific quality

## Abstract

**Background:**

Illumina is the most widely used next generation sequencing technology and produces millions of short reads that contain errors. These sequencing errors constitute a major problem in applications such as *de novo* genome assembly, metagenomics analysis and single nucleotide polymorphism discovery.

**Results:**

In this study, we present ADEPT, a dynamic error detection method, based on the quality scores of each nucleotide and its neighboring nucleotides, together with their positions within the read and compares this to the position-specific quality score distribution of all bases within the sequencing run. This method greatly improves upon other available methods in terms of the true positive rate of error discovery without affecting the false positive rate, particularly within the middle of reads.

**Conclusions:**

ADEPT is the only tool to date that dynamically assesses errors within reads by comparing position-specific and neighboring base quality scores with the distribution of quality scores for the dataset being analyzed. The result is a method that is less prone to position-dependent under-prediction, which is one of the most prominent issues in error prediction. The outcome is that ADEPT improves upon prior efforts in identifying true errors, primarily within the middle of reads, while reducing the false positive rate.

**Electronic supplementary material:**

The online version of this article (doi:10.1186/s12859-016-0967-z) contains supplementary material, which is available to authorized users.

## Background

Error profiles of current high-throughput short read sequencing technologies are different compared with traditional Sanger sequencing [1–3]. Most sequencing technologies come with software that assign quality scores to each nucleotide as a means to estimate the probability of there being an error at that position, and does so by using a measurement (e.g. fluorescence intensity) on the platform. Multiple iterations of calling quality scores has occurred and while the overall quality of a read may be accurate; the scoring systems used fall short when predicting single nucleotide errors. Error detection (and correction) at the single nucleotide may not be a priority for many applications, as high fold coverage over any given nucleotide is generally sufficient to resolve inconsistencies in the data. There are however some applications where error identification may be more essential, such as in the area of metagenomics, where population variation and errors in sequences may be easily confounded.

Current approaches of error detection/correction of reads can be summarized in two categories. One strategy relies on intrinsic properties of the run and corrects erroneous bases without removing them [4–8]. This approach relies on depth of coverage in order to correct errors, by assuming that sufficient depth of coverage will allow identification of errors within the raw data. In this case, low frequency Kmers, a nucleotide sequence of length K, are subject to correction with abundant Kmers that are identical except for 1 position. This does not require quality scores and is good for isolate/single genomes or any clonal genome that is highly covered. This does not work well for low coverage regions, such as low abundance transcripts in RNAseq or for low abundance organisms within metagenomes and will in effect normalize any natural variations in sequence (such as allelic variation) when sequencing populations of highly similar organisms (genomes).

The other strategy removes reads or trims nucleotides based on quality scores. This method can be applied to any sequence, regardless of depth of coverage, since the primary input is the quality score and the cutoff for trimming is a user-defined quality threshold. This is performed from one or both ends of each read, and entire reads can be removed. Commonly used programs such as ConDeTri, SolexaQA, and BWA are implemented using this method [9–11].

In this study, we present A Dynamic Error-detection Program with Trimming (ADEPT), and perform comparisons with these latter tools. ADEPT is an input-specific error detection method that relies on the local quality scores of each nucleotide, as well as its neighboring nucleotides, in comparison with the dataset’s average position-specific scores. We developed our error model based on statistical analysis of errors in Illumina reads. Likely errors within reads are predicted by applying this model to the quality score patterns observed within each specific dataset. As a result, the set of criteria for detecting errors are unique to each read position within any given sequencing run. We show that the incorporation of adjacent nucleotides into an error detection model (and not solely relying on individual nucleotide quality score), greatly improves upon prior efforts, particularly in terms of true positives (i.e. error detection), without increasing the false positive rate. The detected errors are changed to N’s within the read, and a downstream trimming module which allows for both 5′ and 3′ end trimming as well as the optional splitting of reads at N’s (thereby removing identified errors from the reads).

## Implementation

### Overview

We used several Illumina datasets with known reference genomes to establish our model for error prediction, which uses the quality scores of not only the position in question, but also its preceding and following two nucleotides. ADEPT first randomly chooses two hundred thousand reads from raw data input files, and the overall statistics are calculated: the average, minimum, maximum and standard deviation of quality scores for each position over the entire length of the reads. These statistics are unique for each sequencing dataset and establish the baseline for subsequent processing. The statistics are used to assess the likelihood of error for any given position within any read, based on position-specific quality score distributions and incorporates adjacent base quality values.

### Establishing a model for error detection

**Fig. 1:**
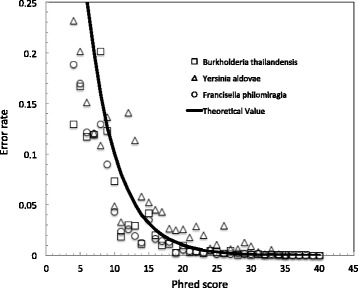
Comparison of predicted error rates with observed error rates. The solid line represents the theoretical, predicted error rate given a Q score, *P = 10^(−Q/10)*, where Q is the Phred quality score and P is the predicted error rate. The actual error rates for all called Q scores are the mean values calculated from all nucleotide positions within all reads for the three datasets: *Burkholderia thailandensis* (*square*), *Yersinia aldovae* (*triangle*), and *Francisella philomiragia* (*circle*). 95 % confidence limits were used as error bars, however, due to the large amount of data sampled, the error bars are too small to be seen, and are covered by the height of the symbol

Primary sequence analysis software included with most sequencing platforms generally provide users with a guide to potential sequencing errors based on a quality scoring system that tries to estimate the probability of any nucleotide being called correctly. We used the read mapping software BWA [5] to align the raw Illumina reads to the finished genomes that were derived from those reads to identify positions within the reads that we consider as real errors. We selected three sequencing datasets and their corresponding genomes from bacteria that represent a range of genomic G + C content: *Burkholderia thailandensis* (68 % G + C), *Yersinia aldovae* (48 % G + C), and *Francisella philomiragia* (33 % G + C). While the general trend observed supports higher quality scores correlating with more accurate base calling, there is yet a large discrepancy between the calculated probability P of accuracy (*P = 10^(−Q/10)*, where Q is the Phred quality score) and the observed experimental value (Fig. 1, Additional file 1: Figure S1). This initial quality scoring provided within Illumina FastQ files appears to over-predict the error rate along the entire spectra of quality scores.

**Fig. 2:**
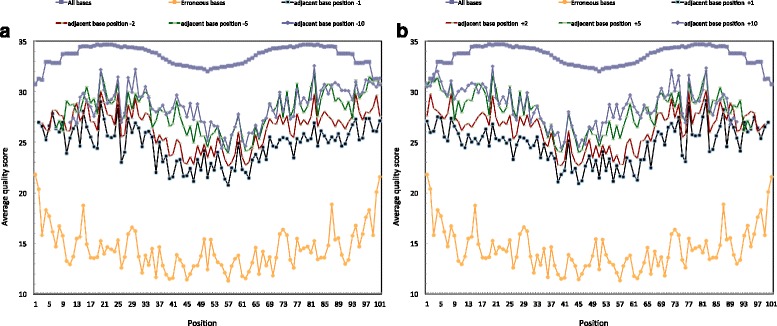
Average quality scores along reads for erroneous bases and their adjacent bases, and for all the reads for *Burkholderia thailandensis*. In 2(**a**), the purple line represents the average quality score of the full Illumina run. The orange line represents the average quality score at erroneous base positions. The other lines represent average quality scores of bases near the erroneous base at positions −1, −2, −5, and −10. In Fig. 2(b), the purple and orange lines are identical to 2(**a**), while the other lines represent average quality scores of bases near the erroneous base at positions +1, +2, +5, and +10

**Fig. 3:**
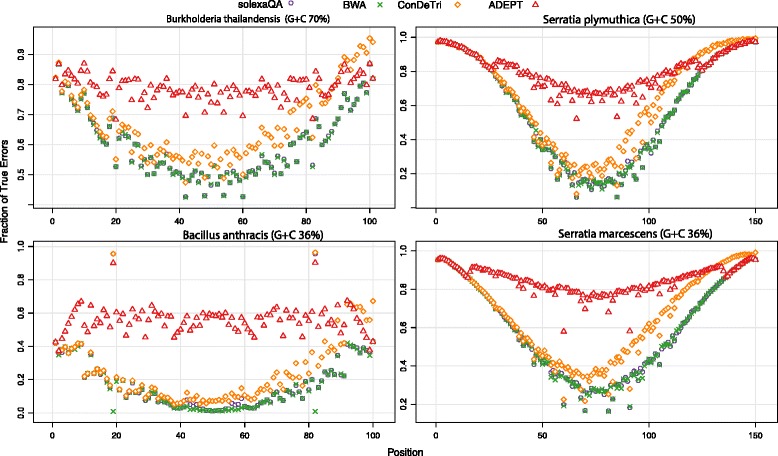
Fraction of known errors identified for diverse samples. The four samples include *Bacillus anthracis* AMES, *Serratia* sp. FGI94, *Burkholderia thailandensis*, and *Serratia plymuthica* RVH1 and are of differing G + C content and/or sequenced on different physical machines. The four methods shown are SolexaQA (*purple circle*), BWA (*green X*), ConDeTri (*orange diamond*) and ADEPT (*red triangle*). Y-axis represents the fraction of the known errors identified by each tool; X-axis represents the position within the reads

Because the quality score is meant to reflect the probability of errors at a given position, we tested whether the position-specific quality scores of correct versus erroneous bases were statistically different, as would be expected. Using the correct versus erroneous position-specific scores, a Wilcoxon Signed-Rank Test clearly showed very small *P*-values on the order of 10^−11^ (i.e. significantly different), over the entire length of the read. Similar *P*-values were also found for at least two positions immediately adjacent (both 5′ and 3′) to the erroneous bases, essentially discriminating the scores of bases adjacent to errors versus those adjacent to correct base calls. Furthermore, comparisons of erroneous base call scores (and adjacent positions) with the scores of the entire dataset (including the erroneous positions) displayed nearly identical Wilcoxon Signed-Rank Test statistics. As an example, Fig. 2 displays a summary of the quality scores over the entire length of all reads for *Burkholderia thailandensis*, showing correct bases, erroneous bases, and the bases adjacent to erroneous base calls. The sequence errors on average have much lower quality scores than correct base calls. Furthermore, the positions adjacent to the erroneous base calls also have lower quality values than might otherwise be expected for a correct base call, both 5′ (Fig. 2a) and 3′ (Fig. 2b). Similar trends were found with the two other projects despite their very different G + C content, *Yersinia aldovae* and *Francisella philomiragia*, and are presented in the Additional file 1: Figure S2 and Figure S3, respectively. Based on these findings, we sought to improve upon traditional error detection/trimming methods that only use a user-defined quality score threshold for all positions along the entire read.

### Algorithm

Based on the above observations, we reasoned that the position-specific quality distribution should be accounted for over the entire length of the reads, and adjacent base quality values should be taken into account when assessing errors. Here we present a method that takes this local and position-specific information into account, and that can accommodate sequencer, or even run-to-run variations. Therefore, ADEPT’s underlying algorithm is based on the observed differences of Q scores between the erroneous bases (and local adjacent bases) and the corresponding position-specific scores of the entire dataset.

The software was written in Perl5.8 and is executed as a command line tool. The only input files required by ADEPT are one or more FASTQ files (either single end or paired-end reads). ADEPT utilizes the Parallel::ForkManager Perl module to allow parallel processing and to control sub-processes. The input dataset is initially split by default into multiple files of four million reads each, but users can tune this parameter via a command line flag. Each split file is independently run through the ADEPT process, controlled by ForkManager in parallel.

ADEPT investigates reads in three sequential steps:

The *first* step uses a traditional method of identifying likely errors at both the 5′ and 3′ ends of reads, since the ends often have poor quality. We implemented a sliding window-based approach [12] to identify these errors using default parameters. The nucleotides at these positions are converted to Ns for downstream processing.

The *second* step randomly samples up to 10 million reads from a given input dataset to automatically establish the baseline distribution of quality scores and determines the parameter settings for the model of error prediction of this particular sequencing dataset. This concept of using the intrinsic qualities of the data as a guide for error-detection is unique and not found in other programs. ADEPT then investigates all reads and bases that pass the first step and identifies a nucleotide as correct if the quality score is above the median score for that position within the sampled run (i.e. higher than 50 % of the quality scores at that position). This threshold is a tunable parameter, should the user decide to be more stringent or lenient in the automatic calling of a correct base. For example, changing this parameter to 40 % would allow 10 % more bases to be automatically identified as correct. In addition this step can include automatically identifying a nucleotide as an error if it falls below a defined percentage of the quality scores for that position (this parameter is set to 0 % as a stringent default, meaning that no nucleotide is ever identified automatically as an error). For example, setting this threshold to 5 % would essentially guarantee calling 5 % of the bases as incorrect for any given position. Those identified as incorrect are converted to Ns for downstream processing.

The *third* step incorporates the quality values of adjacent nucleotides for all positions (that are not already identified as correct or as errors in step 2). In this step, ADEPT uses two criteria that include using the scores of adjacent base positions.

The first criterion is determined by the ratio of the base quality to the qualities of *n* upstream and downstream positions, *R*_*in*_ = *Q*_*i*_/*Q*_*i* ± *n*_, where Q_i_ is the quality score at the *i*th position and *Q*_*i* ± *n*_ are the quality scores at positions *i* ± *n*. By default, all R_in_ ratios must be smaller or equal to 0.4 to be considered as a potential erroneous base (i.e. all adjacent qualities must be at least 2.5 times higher than the quality of the position being investigated for that position to be considered as a potential error). Relaxing the stringency of R_in_ (i.e. higher than 0.4) would consider more bases as potential errors.

The second criterion is determined by the position of *Q*_*i* ± *n*_ within the distribution of all quality values for the *i* ± *n* positions. By default, the quality of the *i* ± *n* positions must all be within the bottom 30 % of the distribution of quality values for that position to continue to be considered as a potential error. Increasing this cutoff above 30 % will have the effect of including more bases as potential errors. Only positions that satisfy all these criteria are identified as erroneous bases, which are then converted to Ns for downstream processing.

We examined the effect of using adjacent bases in the identification of errors and compared the results of using only the quality score of the erroneous base with incorporating the qualities of either one, two or three adjacent bases, or using a randomly selected base from within the read (Additional file 1: Figure S4). We found that including adjacent positions to the base of interest offers substantial error-identification improvement over the simple use of the quality score of the base itself. Additional file 1: Figure S4 also shows that including two adjacent positions performs better than only a single adjacent position (primarily in the middle of the read) and that including additional adjacent positions beyond two did not notably increase the performance of finding errors. Therefore, to accommodate computational efficiency, ADEPT considers only two upstream and two downstream positions.

After the determination of where errors reside, trimming occurs to remove identified errors. To maximize efficiency, this trimming occurs as the reads are processed. By default, ADEPT trims any continuous stretches of N’s at the 5′ and 3′ ends from reads and keeps the N’s in the middle of reads but changes the quality scores to zero. All remaining reads greater than a user-specified minimum length (50 bp default) are placed in a new FASTQ output file. For paired-end reads, if only one read of a pair is retained, it is placed within a separate FASTQ file that contains unpaired reads. Users also have an option to trim the N’s in the middle of reads. This option splices reads at any N, and retains only the longest remaining fragment for the new FASTQ output file (subject to the same user-specified minimum length). Users can also output untrimmed reads, where the identified errors are replaced by Ns (with quality scores of zero).

## Results and discussions

We evaluated ADEPT and compared its performance in identifying errors with three other tools (ConDeTri, SolexaQA, and BWA) using four independent datasets (see Additional file 1: Table S1) that have finished genomes, namely, *Bacillus anthracis Ames_BA1004*, S*erratia marcescens* FGI94, *Burkholderia thailandensis* 2002721723, and *Serratia plymuthica* RVH1. These four datasets span a wide range of G + C (36-70 %), read length (100-150 bp reads), and were generated on different sequencers and even at different genome centers. When run on an eight-core machine (Intel(R) Xeon(R) CPU X5675 @ 3.07GHz), ADEPT requires approximately one hour per 15 million reads and a maximum of ~20GB memory to process the entire dataset.

True errors were identified by comparing all reads to the finished genomes. The percentage of true errors identified by each method is shown in Fig. 3. While the performance of the tools is somewhat similar at the 5′ and 3′ ends of reads, ADEPT excels in identifying true errors in the middle of the reads for all datasets and outperforms the other tools. This improvement appears independent of G + C content, read length or sequencer used. We note that ConDeTri, a content dependent read trimmer did outperform the two other tools, particularly in the second half of the reads. In almost all cases, SolexaQA, ConDeTri, and BWA identify 20-60 % fewer true errors in the middle portion of the read (~50 % of the read length) and display a concave distribution of identified true errors along the length of the reads. This curvature is less pronounced with ADEPT, indicating substantially improved position-independent error detection. We also note that the proportion of true errors found can differ substantially (with all tools) depending on the dataset.

**Fig. 4:**
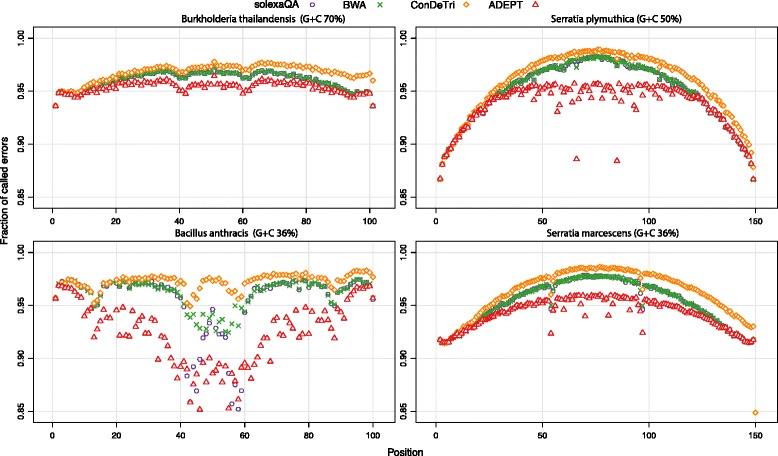
The fraction of incorrectly identified errors for diverse samples. The four samples include *Bacillus anthracis* AMES, *Serratia* sp. FGI94, *Burkholderia thailandensis*, and *Serratia plymuthica* RVH1 and are of differing G + C content and/or sequenced on different physical machines. The four methods shown are SolexaQA (*purple circle*), BWA (*green X*), ConDeTri (*orange diamond*) and ADEPT (*red triangle*). Y-axis represents the fraction of called errors that are incorrect (i.e. the tools called these errors but they are correct bases); X-axis represents the position within the reads

While identification of additional true errors is important, we also wanted to investigate if this was solely due to calling many more predicted erroneous positions. Figure 4 displays the fraction of false positives to the total errors called (i.e. fraction of positions called as erroneous but that are not errors). In all cases and all sequence positions, the fraction of false positive errors ranges from 85 to 99 %, indicating that most of the predicted errors are in fact not erroneous, and by extension, that there are many accurately-called nucleotides in any given sequencing run are assigned low quality scores. This corroborates our findings in Fig. 1, indicating that the quality scores do not always reflect true error probabilities. However, this proportion is consistently lower throughout the entire length of the read when using ADEPT compared with the other methods. This improved performance over other programs (reduced false positive rate, Fig. 4; and improved true positive rate, Fig. 3) is more prominent in the middle of the reads than the 5′ or 3′ ends. As shown in Additional file 1: Figure S5, the fraction of false errors called compared with the total reads at a given position can be substantially high, and in some cases can be as much as ~40 % at the ends of reads. The extent of these erroneous predictions varies depending on the dataset and the quality of the run, as all tools use the native quality scores to predict errors. While this may dissuade researchers from using such tools with high false positive rates, the high sequencing throughput combined with the elimination of most errors generally provide improved results after using these error-prediction and trimming tools.

Using another independent dataset, we tested and compared the effect of ADEPT with other tools, and specifically looked at Velvet assembly results compared with untrimmed data (Additional file 1: Table S2). Using Velvet (Version 1.2.08) with *K* = 77, ADEPT offers improvements in terms of contig N50 and maximum contig size. This improvement and favorable comparison with the other tools, comes despite the fact that Velvet cannot take advantage of ADEPT output (neither the N’s replacing the suspected erroneous bases, nor their qualities set to zero). Assembly and other analysis tools that are used downstream of error-detection algorithms, would need to be reconfigured in order to take full advantage of ADEPT output.

## Conclusions

Here we present ADEPT, a tool that dynamically assesses errors within reads based on position-specific and local quality scores. It is the first tool that we are aware of that dynamically processes data and relies on within-dataset information to identify errors. The method used to devise the error model for Illumina data can readily be applied for assessing and detecting errors in other technologies. The key to ADEPT is the analysis of quality scores not only of the base being analyzed, but also the scores of its neighboring bases, and how these relate to the entire dataset in a position-specific fashion. ADEPT outperforms other tools with respect to identifying true errors without increasing the total errors called. This is particularly true within the middle of reads, because other tools rely almost exclusively on the quality scores of the base being considered, and because these scores are typically poor at the ends of reads, and their inability to distinguish errors in the higher quality middle portion of reads. Taking into account position-specific scores, neighboring base scores, and relating these to the distribution of scores in a position-specific manner provides ADEPT with a superior true positive error rate. The ability to identify errors within the middle of reads may be particularly important in the case of metagenomic data analysis when the genome coverage may be very low. Perhaps more importantly, the methodology presented here provides a framework that can be extended to other sequencing technologies.

## Availability and requirements

Project name: ADEPT

Project home page: https://github.com/LANL-Bioinformatics/ADEPT

Operating system(s): Platform independent with primary UNIX support

Programming language: Perl and R

Other requirements: Perl Parallel::ForkManager from CPAN http://search.cpan.org

License: GNU GPL version 3 or later

## Additional file

Additional file 1: Supplemental file. (PDF 600 kb)
