# Supplementary material for: ADEPT, a dynamic next generation sequencing data error-detection program with trimming
Source: BMC Bioinformatics. 2016 Feb 29;17:109. doi: 10.1186/s12859-016-0967-z (PMC4772517; doi:10.1186/s12859-016-0967-z)

Supplementary

Table S1. Statistics for genomes and sequencing datasets used in this study.

Name	Sequence length	G+C	Read length	# of pair reads	SRA	Accession #
Burkholderia thailandensis 34[1]	3,896,054	68%	101	42,950,955	SRX498885 [a]	CP010017.1
Yersinia aldovae 670-83[1]	4,471,088	48%	101	30,025,126	SRX869060 [a]	CP009781.1
Francisella philomiragia[1]	2,017,393	33%	241	13,997,096	SRX1159901[a]	CP010019.1
Bacillus[1] *anthracis Ames BA1004*	5,503,972	36%	100	36,679,066	SRX856499 [a]	CP009981.1
Burkholderia[1] *thailandensis 2002721723*	6,577,133	70%	101	47,883,213	SRX729936 [a]	CP004097.1
Serratia plymuthica RVH1[1]	5,514,320	50%	150	34,899,793	Gp0012814[b]	ARWD01000001.1
Serratia marcescens FGI94[1]	4,858,216	36%	150	8,989,515	Gp0009213[c]	CP003942.1
Burkholderia dolosa AU0158[2]	6,420,400	67%	101	18,019,832	SRX1134792[a]	CP009795.1
Francisella philomiragia O#319L[3]	2,017,400	33%	101	2,797,931	SRX869092[a]	CP010019.1

a. available at https://www.ncbi.nlm.nih.gov/
b. available at https://gold.jgi.doe.gov/project?id=Gp0012814
c. available at https://gold.jgi.doe.gov/project?id=Gp0009213
1. HiSeq, 2000, RTA 1.12, created in 2011.
2. HiSeq, 2000, RTA 1.12.4.2, created in 2015.
3. MiSeq, RTA 2.4.6, Created in 2015.

Table S2. Statistic of Burkholderia dolosa AU0158 (G+C 67%) assembly using Velvet (Version 1.2.08) with k=77.

Trimming method	Total number of reads	Average read length	# Contigs	Fold coverage	N50	Maximum Contig Length	Total Number of Bases
Untrimmed	36039664	101	243	577.4	55441	192731	6303897
ConDeTri	20536052	98.3	209	319.8	64491	197625	6312680
BWA	36039664	95.4	200	545.2	77855	267319	6306591
SolexaQA	36039664	81.1	192	463.2	71843	197625	6311857
ADEPT	33272274	98.7	205	520.7	78943	267319	6306405

Figure S1. Comparison of predicted error rates with observed error rates. The solid line represents the theoretical, predicted error rate given a Q score, *P=10^(-Q/10)*, where Q is the Phred quality score and P is the predicted error rate. The actual error rates for all called Q scores are the mean values calculated from all nucleotide positions within all reads for the two datasets: *Burkholderia dolosa* (diamond), and *Francisella philomiragia* (triangle). 95% confidence limits were used as error bars. Due to the large amount of data sampled, the error bars are small and covered by the height of the symbol.

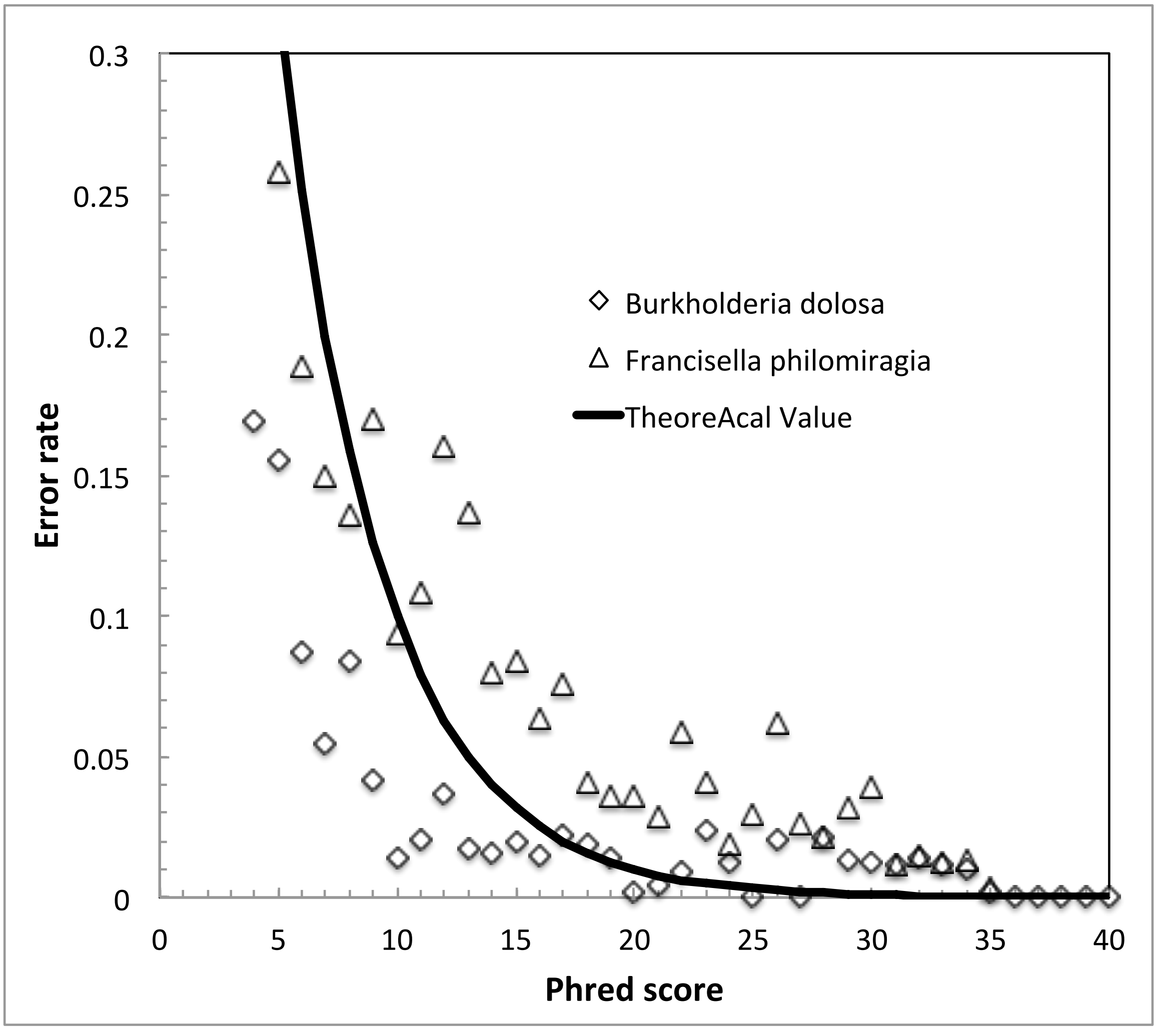

Figure S2a: Average quality scores along reads for erroneous bases and their adjacent bases, and for all the reads for *Yersinia aldovae*. The purple line represents the average quality score of the full Illumina run. The organe line represents the average quality score at erroneous base positions. The other lines represent average quality scores of bases near the erroneous base at positions -1, -2, -5, and -10

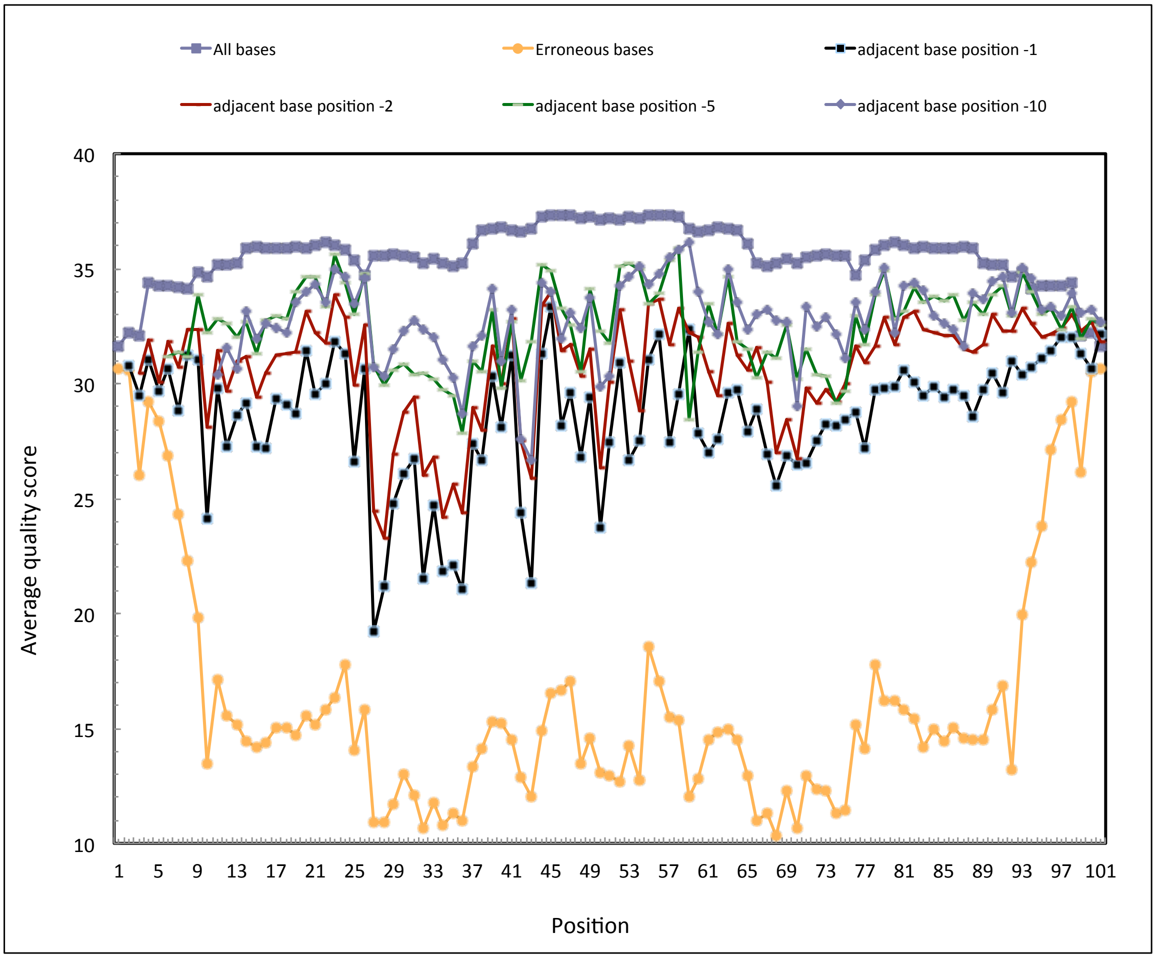

Figure S2b: Average quality scores along reads for erroneous bases and their adjacent bases, and for all the reads for *Yersinia aldovae*. The purple line represents the average quality score of the full Illumina run. The organe line represents the average quality score at erroneous base positions. The other lines represent average quality scores of bases near the erroneous base at positions +1, +2, +5, and +10.

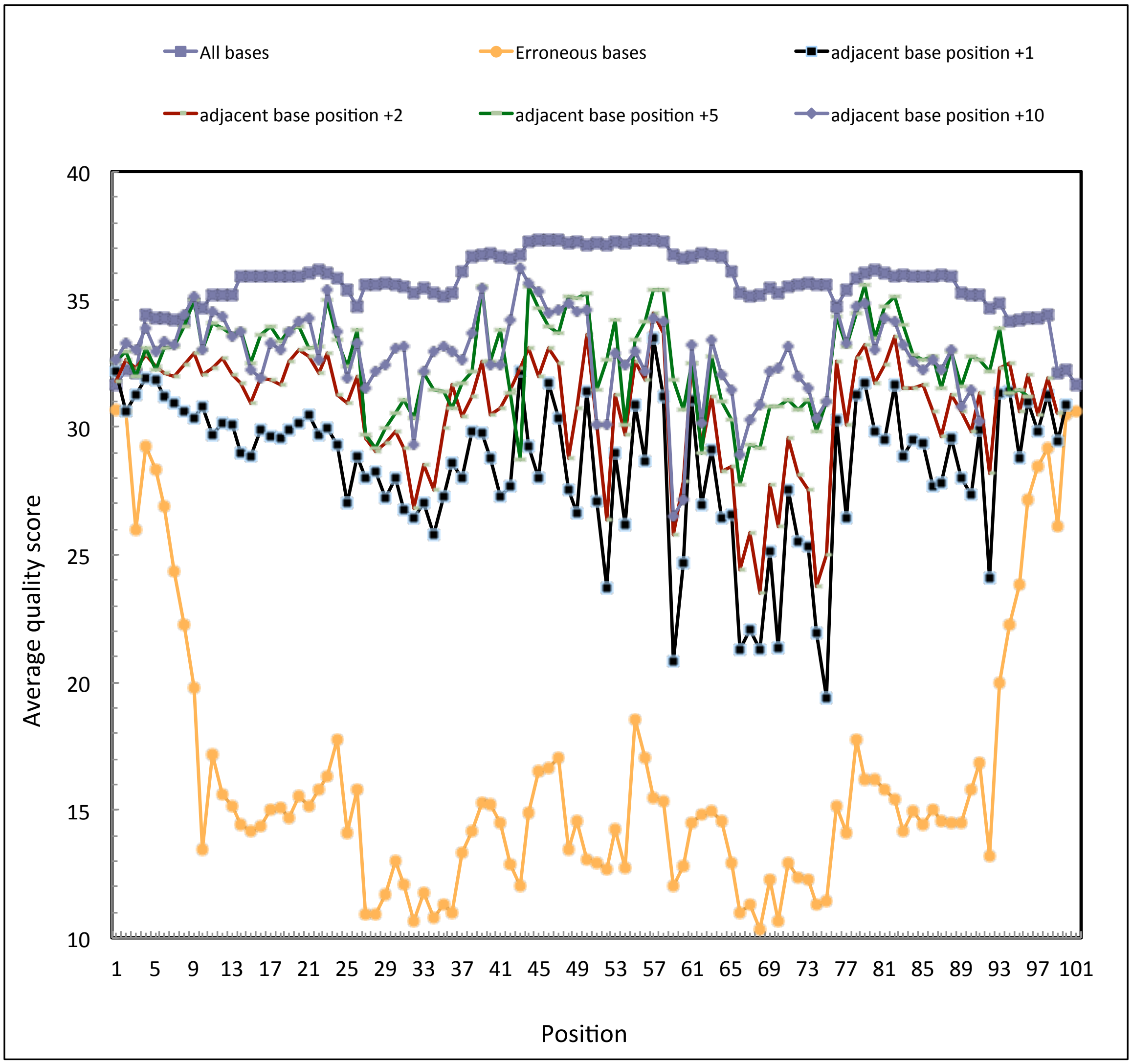

Figure S3a: Average quality scores along reads for erroneous bases and their adjacent bases, and for all the reads for *Francisella philomiragia*. The purple line represents the average quality score of the full Illumina run. The organe line represents the average quality score at erroneous base positions. The other lines represent average quality scores of bases near the erroneous base at positions -1, -2, -5, and -10.

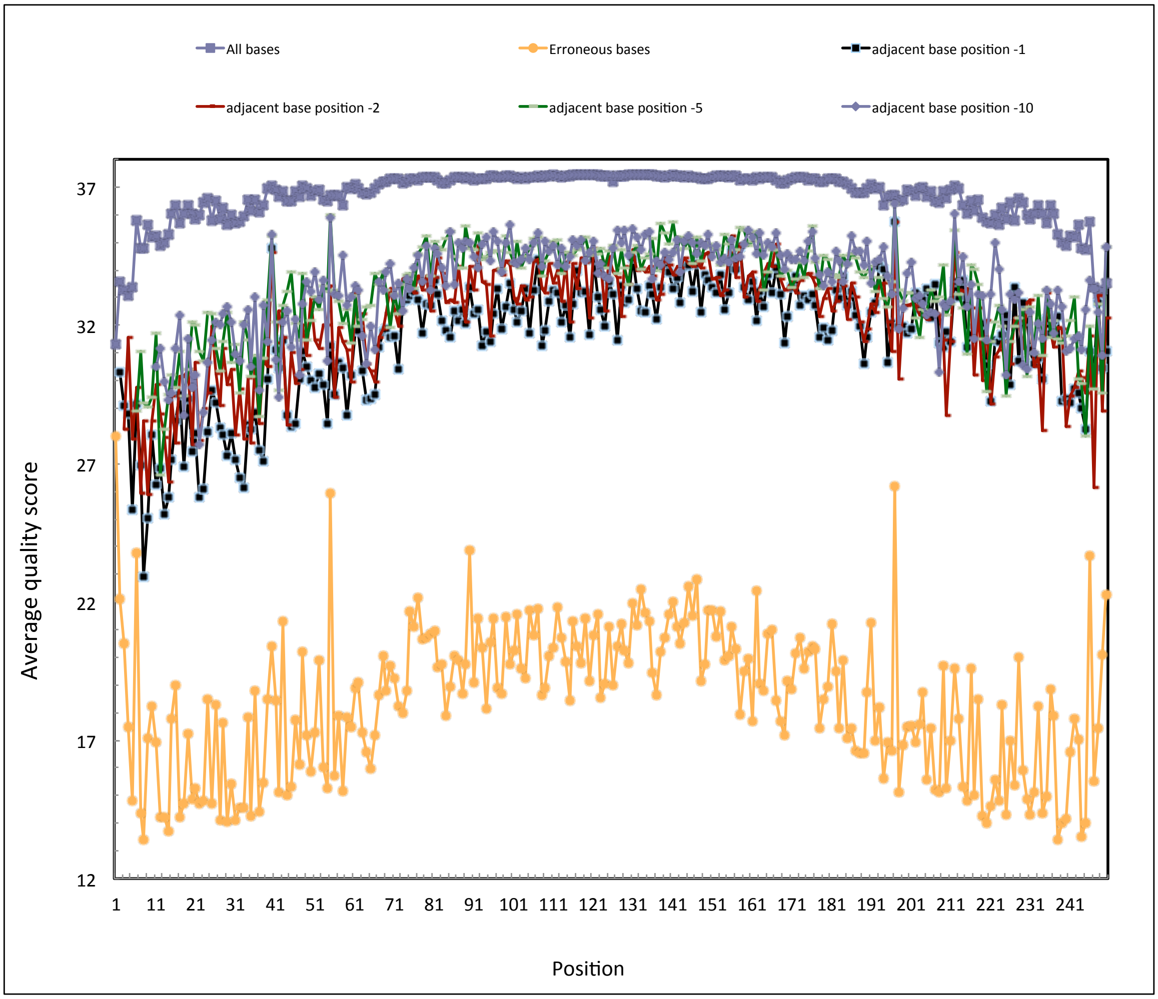

Figure S3b: Average quality scores along reads for erroneous bases and their adjacent bases, and for all the reads for *Francisella philomiragia*. The purple line represents the average quality score of the full Illumina run. The organe line represents the average quality score at erroneous base positions. The other lines represent average quality scores of bases near the erroneous base at positions +1, +2, +5, and +10.

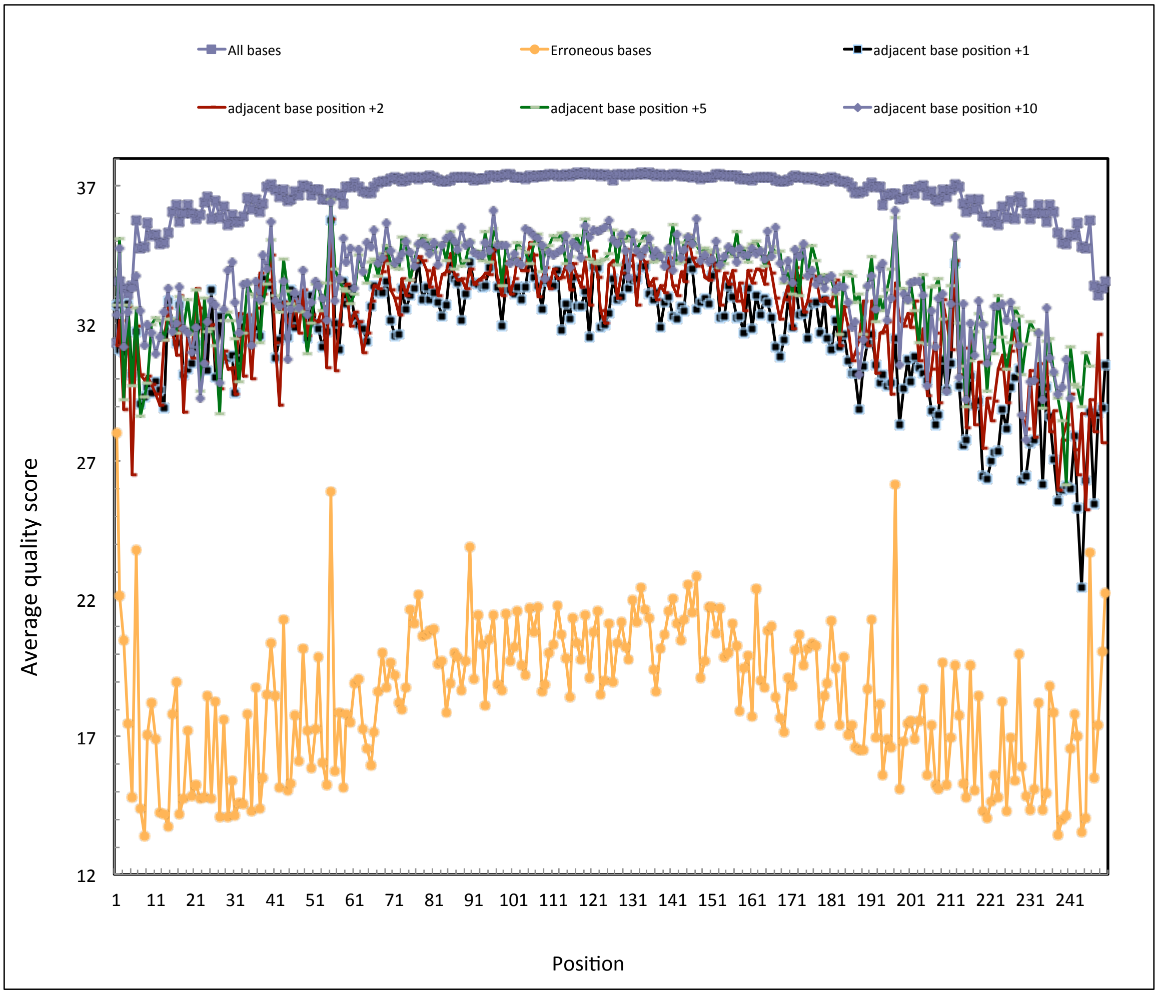

Figure S4. Fraction of known errors identified for Bacillus anthracis (G+C 36%) using five different methods. The five samples include using only the QC (Phred score) at the base considered (square), using QC at the base considered plus one adjacent upstream/downstream bases(triangle), using QC at the base considered plus two adjacent upstream/downstream bases(circle), using QC at the base considered plus three adjacent upstream/downstream bases(diamond), and using QC at the base considered plus one a random base(star).Y axis represents the fraction of the known errors identified by method; X axis represents the position within the reads.

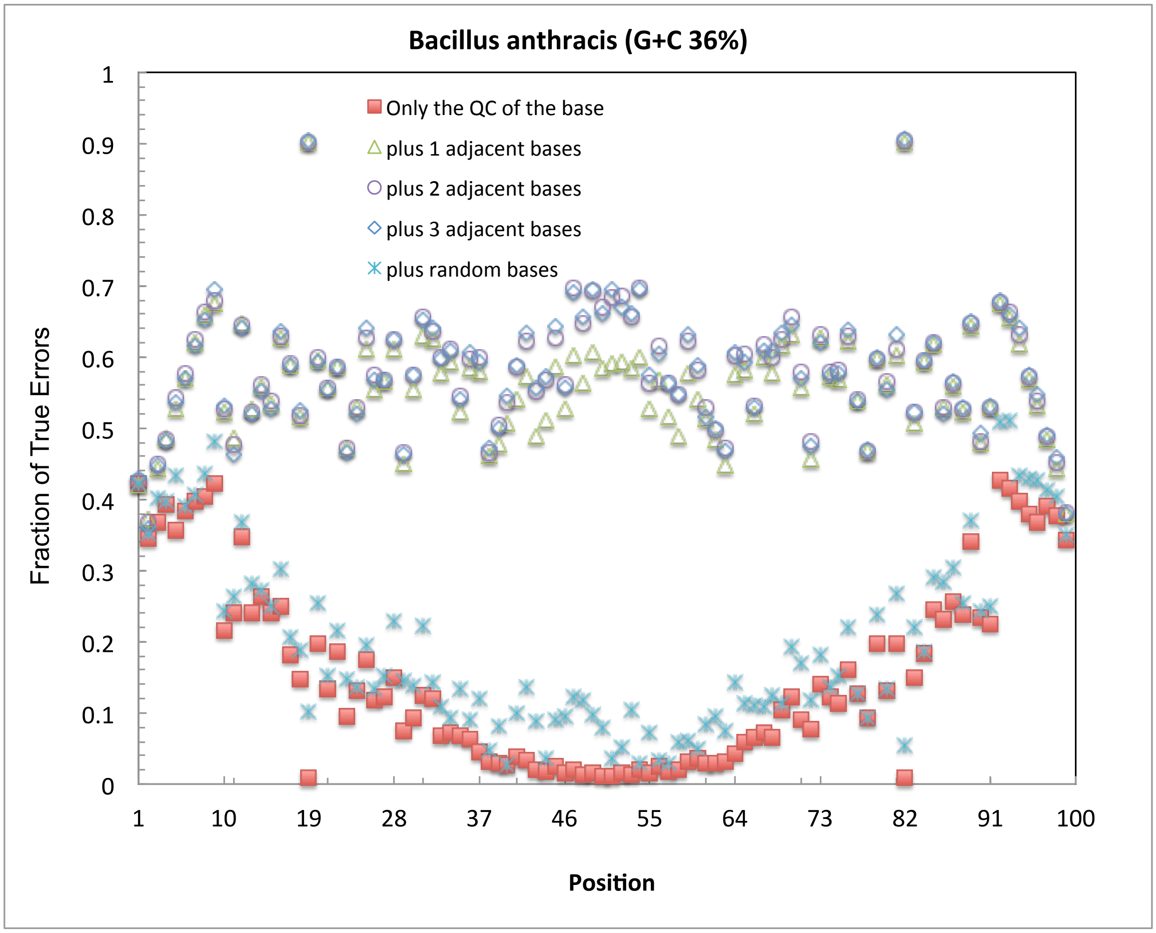

Figure S5. The ratio of false errors identified to the total number of reads by different methods at each base position The four samples include Bacillus anthracis, Serratia plymuthica, Burkholderia thailandensis, and Serratia plymuthica that represent different range of GC contents. SolexaQA method shown as circle, BWA method shown as cross, ConDeTri shown as diamond and ADEPT method shown as triangle. The x-axis represents the position of the base. The y-axis represents the ratio of false errors identified to the total number of reads.

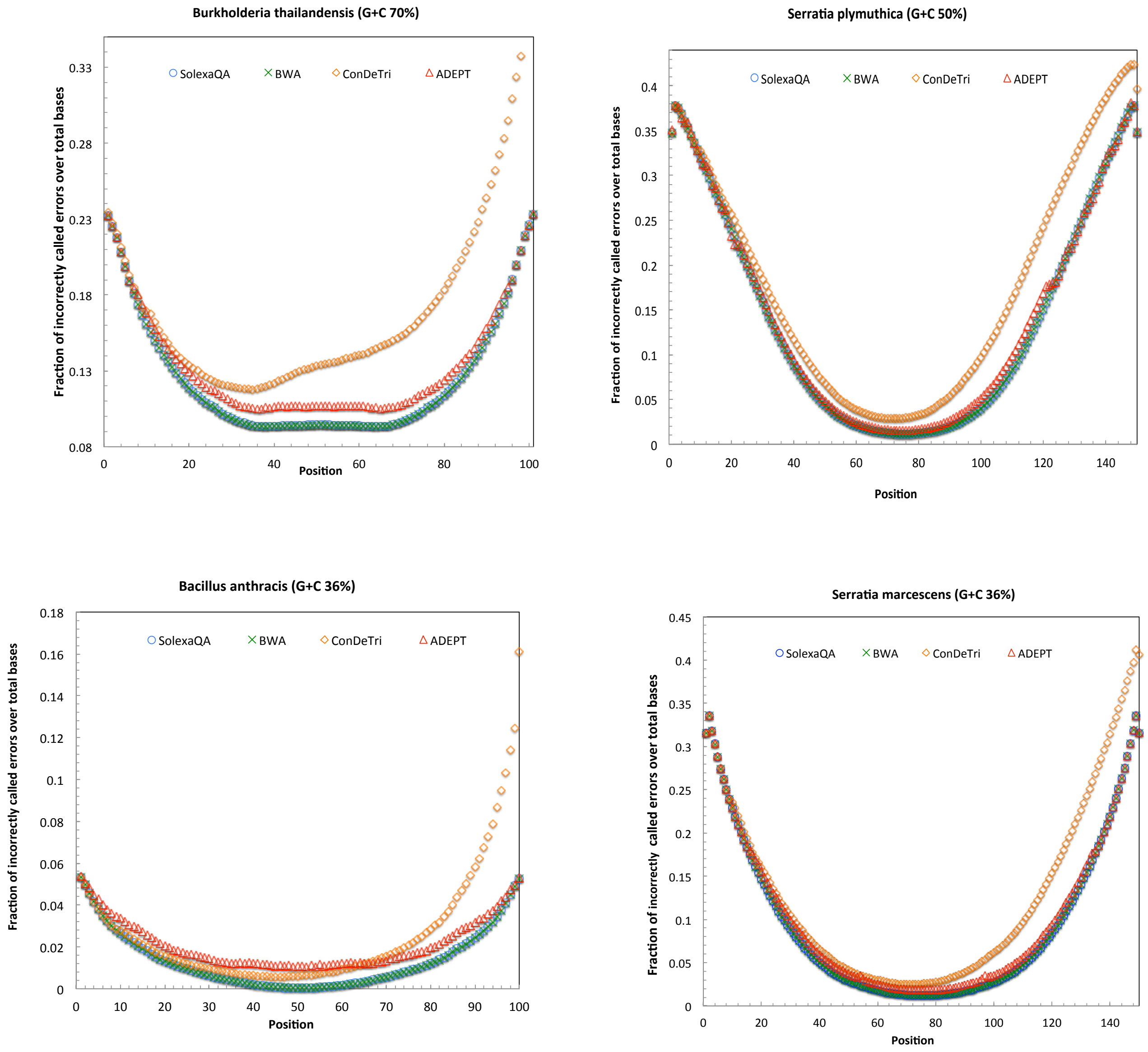

Supplement: Additional file 1: — Supplemental file. (PDF 600 kb) [file 12859_2016_967_MOESM1_ESM.pdf]
